# Conduct problems and callous‐unemotional traits in pre‐schoolers: Psychosocial profiles in a Brazilian birth cohort

**DOI:** 10.1111/jcpp.70164

**Published:** 2026-05-16

**Authors:** Luciana Rodrigues Perrone, Andreas Bauer, Rebecca Waller, Marlos Domingues, Pedro Curi Hallal, Joseph Murray

**Affiliations:** ^1^ Postgraduate Program in Epidemiology Federal University of Pelotas Pelotas Rio Grande do Sul Brazil; ^2^ Human Development and Violence Research Centre (DOVE) Federal University of Pelotas Pelotas Rio Grande do Sul Brazil; ^3^ Department of Preventive Medicine, Faculty of Medicine University of São Paulo São Paulo Brazil; ^4^ Department of Psychology University of Pennsylvania Philadelphia PA USA; ^5^ Postgraduate Program in Physical Education Federal University of Pelotas Pelotas Rio Grande do Sul Brazil; ^6^ Department of Kinesiology and Community Health University of Illinois Urbana‐Champaign Urbana‐Champaign IL USA

**Keywords:** Callous‐unemotional traits, conduct problems, early childhood, psychosocial characteristics

## Abstract

**Background:**

Callous‐unemotional (CU) traits in early childhood are risk markers for persistent antisocial behaviour, with marked adverse life outcomes. However, associations of CU traits with broader psychosocial characteristics are not well understood, particularly in early childhood.

**Objective:**

To examine developmental and psychosocial profiles of pre‐school children according to their levels of CU traits and conduct problems (CPs) in a large, population‐based cohort from southern Brazil.

**Methods:**

The 2015 Pelotas Birth Cohort includes 4,275 children followed from birth. At age 4 years, CU traits and CP were assessed via parent‐report questionnaires, and 19 other psychosocial characteristics were examined mostly in task‐based assessments regarding: neurodevelopment, language, executive functioning, emotion recognition, theory of mind, social information processing and empathy/prosocial behaviours. These 19 psychosocial characteristics were compared across four groups of children with varying levels of CU/CP, and regression models were used to test for independent associations with CU traits and CP, adjusting for sociodemographic factors at birth.

**Results:**

At age 4 years, 14.8% of children had elevated CU traits only, 14.2% had CP only, and 16.3% had both high CU + CP. Compared to children without CU traits or CP, children with higher CU traits had poorer performance across multiple domains of development, including language, motor development, emotion recognition and prosocial behaviour. In contrast, children with CP‐only showed more circumscribed deficits, mainly in self‐control and emotional functioning. In regression analyses, CU traits were associated with broader neurodevelopmental impairments, whereas CP was more specifically linked to behaviour regulation.

**Conclusions:**

Elevated CU traits in early childhood are associated with broad developmental difficulties, whereas CPs relate more specifically to emotional and self‐regulation challenges. These findings underscore the need for holistic approaches to understanding early manifestations of CU traits and interventions in the context of CP behaviours.

## Introduction

Callous‐unemotional (CU) traits reflect a temperamental dimension marked by reduced empathy, limited prosociality and shallow emotional responsiveness (Waller et al., 2020; Waller & Hyde, 2018). Considerable research and clinical interest in CU traits has been motivated by evidence that childhood conduct problems (CPs) are both more frequent and severe when accompanied by CU traits (Blair, Leibenluft, & Pine, 2014; Waller et al., 2016), and that this pattern is strongly predictive of adverse social, physical and health consequences into adulthood, including outcomes of substance abuse, crime, unemployment and early death (Moffitt, 2018; Squillaci & Benoit, 2021; Wilson et al., 2013). As such, CU traits have been incorporated as a specifier for a sub‐type of conduct disorder ‘with Limited Prosocial Emotions’ in both major mental health diagnostic systems, to help identify children with CPs with greater challenges and poorer prognoses (DSM‐5 and ICD‐11). The same specifier was included for oppositional defiant disorder in ICD‐11 (Deng et al., 2024). However, an important question remains about whether challenges for young children with elevated CU traits and CPs are specific to those behavioural‐emotional domains, or whether they tend to manifest a broader range of developmental delays, implying a more holistic approach both for theoretical understanding and treatment.

Several studies have found that children presenting high CPs and CU traits exhibit significant concurrent differences in socioemotional, cognitive and biological functioning, compared to children only manifesting high CPs (Frick & Ray, 2015). Key findings show that children with CU traits tend to have more difficulties with emotion recognition (Sharp, Vanwoerden, Van Baardewijk, Tackett, & Stegge, 2015), lower empathy (Georgiou, Kimonis, & Fanti, 2019) and poorer social information processing (Helseth, Waschbusch, King, & Willoughby, 2015). However, a thorough delineation of how CU traits relate to these phenomena is lacking, particularly outside clinical contexts. Moreover, the question of whether broader aspects of child development, such as broad neuropsychomotor development, language and executive functions, are also related to CU traits has received little research attention. If CU traits do relate to other developmental challenges, a key issue is whether that is principally because of the association between CU traits and CPs, or whether CU traits themselves might independently predict other developmental challenges. Population‐based samples are needed to delineate children with full variation in both CU traits and CPs to advance such understanding, but most existing evidence is based on clinical samples.

Although CU traits can be identified in children as young as 2 years (Blair et al., 2014), and early childhood represents a period of greater potential for change before behavioural problems become entrenched, most research on CU traits has focussed on mid‐late childhood (Northam & Dadds, 2020). Another gap in the evidence base is that the intersection between CU traits and CPs has been researched almost entirely in high‐income country settings (Ritchie, Neufeld, Yoon, Li, & Mitchell, 2022). In the Global South, distinct environmental stimuli may alter child development in ways significantly related to CU traits and CPs. Moral contexts, shaped by social and cultural environments, influence how children learn to express or suppress behaviours such as empathy and aggression through internalised norms, values and expectations (Miller, Bersoff, & Harwood, 1990). Hence, new research is needed to understand the range of psychosocial challenges related to CU traits and child CPs, particularly in early childhood, and in settings in the Global South.

This study aims to examine how CU traits and CPs are associated with a broad range of developmental characteristics in preschool children from a large, population‐based, Brazilian birth cohort. Specifically, we examined children's neuropsychomotor development, language and executive functioning as well as emotion recognition, theory of mind, social information processing and prosocial behaviours, according to their levels of CU traits and CPs. These characteristics were analysed both jointly and independently to capture their distinct and overlapping contributions to children's developmental profiles. Based on developmental and psychopathological models of early antisocial behaviour, we hypothesised that children with typical levels of both CU traits and CPs would show the most favourable outcomes, whereas those with elevated CU traits and CPs would show the poorest outcomes. We further hypothesised that children with elevated levels specific to only one dimension (either CPs or CU traits) would display intermediate developmental profiles, reflecting graded psychosocial risk in early childhood.

## Methods

### Study design and participants

The 2015 Pelotas Birth Cohort is an ongoing, population‐based, prospective longitudinal study investigating a variety of determinants of health and social outcomes. All children born to women residing in the urban area of Pelotas city in southern Brazil in 2015 were eligible to join the study. Throughout that year, daily visits were made to all maternity hospitals (over 99% of children in Pelotas are born in hospitals), and of 4,333 eligible children, 4,275 were enrolled in the study (98.7% response rate; 49.4% girls). Of those, 4,010 were assessed at the age of 4 years, with a follow‐up rate of 95.4% (those assessed and located but died), when the main measures for the current study were collected. Among these, 3,997 had valid data on measures of both callous‐unemotional (CU) traits and CPs and were included in the current analyses. At 6–7 years, 3,867 participants were assessed, with a follow‐up rate of 92.0%. Trained interviewers conducted assessments primarily in the research clinic, using RedCap for data collection (Harris et al., 2009). For more detailed information on the cohort, see Hallal et al. (2018) and Murray et al. (2024).

### Measures

#### CU traits

CU traits were measured at age 4 years using the 12‐item Inventory of Callous‐Unemotional Traits–Short Form (SF‐ICU; Hawes et al., 2014), which assesses child empathy and guilt, insensitivity to others' feelings and shallow emotions. Parents rate how well each item describes their child, with no defined recall period, with items scored on a 4‐point Likert scale (ranging from 0 = *not at all true* to 3 = *definitely true*). The final sum score ranges from 0 to 36 points, with higher scores representing higher CU traits. In the absence of pre‐defined cut‐points, we identified children with scores of ≥10 as having higher levels of CU traits (see Analysis Strategy for details on the ROC curve analysis to identify this cut‐point, and sensitivity analyses based on an alternative).

#### Conduct problems

CPs were measured at age 4 years using the CP sub‐scale of the parent report version of the Strengths and Difficulties Questionnaire (SDQ), which includes five items about behavioural problems in the past 6 months (Goodman, 1999), with items scored on a 3‐point Likert scale (0 = *false*, 1 = *more or less true* and 2 = *true*) producing a total score ranging from 0 to 10. Following the SDQ scoring manual, we used a cut‐off value of ≥4 to identify children with higher levels of CP.

#### Conduct disorder

The Development and Well‐Being Assessment (DAWBA) was used to generate psychiatric diagnoses at the 6–7 years follow‐up, for use in ROC analyses regarding CU traits. DAWBA is applied in interviews with caregivers to generate ICD‐10 and DSM‐IV‐TR or DSM‐V diagnoses among 2–17‐year‐olds (Goodman, Ford, Richards, Gatward, & Meltzer, 2000). DAWBA was applied by trained psychologists, and two psychologists carried out the rating process after data collection. In ROC analyses, we used an indicator for disruptive behaviour disorder, defined by children having either conduct disorder or oppositional defiant disorder (vs. neither disorder).

#### Psychosocial evaluations

The following psychosocial characteristics of children were examined at age 4 years to describe profiles of children according to levels of CU traits and CPs: neuropsychomotor development, language, executive functions, emotion recognition, theory of mind understanding, social information processing and prosocial behaviour. Sub‐dimensions were also assessed for several of these broad constructs, including three specific aspects of social information processing: hostile attribution bias, aggressive response generation and competent assessment. The instruments used to measure all 19 psychosocial characteristics are summarised in Table 1, and more detailed information is in the Appendix S1.

**Table 1:** Psychosocial characteristics examined children's callous‐unemotional traits and conduct problems in the 2015 Pelotas Birth Cohort Study

Broad construct	Specific construct	Instrument	Score range
Neuropsychomotor development	Personal‐social Adaptative Fine and gross motor Communication Cognitive	Battelle's Development Inventory (Barros, Matijasevich, Santos, & Halpern, 2010)	0–28 0–28 0–28 0–24 0–24
Language	Expressive vocabulary	TVexp‐100 (Capovilla, Negrão, & Damázio, 2011)	0–100
	Receptive vocabulary	TVAud‐33 (Capovilla et al., 2011)	0–33
Executive functions	Self‐control	Interviewer Rating Child Self Control (Caspi, Henry, McGee, Moffitt, & Silva, 1995)	4–12
	Gratification delay	Marshmallow Task (Watts, Duncan, & Quan, 2018)	0–210
	Impulse control	Early Years Toolbox Go/No‐Go (Howard & Melhuish, 2017)	0–1
	Attention shifting	Early Years Toolbox Card Sorting (Zelazo, 2006)	0/1
Emotion recognition/Theory of mind	Emotion recognition	Affect Knowledge Test (Denham et al., 2014)	0–8
	Theory of Mind understanding	Sally‐Anne test (Fujino, Fukushima, & Fujiyoshi, 2017)	0–3
Social information processing	Hostile attribution bias	Social Information Processing Interview (Ziv & Sorongon, 2011)	0/1
	Aggressive response generation		0/1
	Competent assessment score		0–9
Prosocial behaviour	Empathy and altruism	Help task (Buttelmann, Carpenter, & Tomasello, 2009)	0–3
	Altruism	Dictator game (Benenson, Pascoe, & Radmore, 2007)	0–10
	Prosocial behaviour	SDQ – prosocial behaviour scale (Goodman, 1999)	0–10

#### Confounding variables

The following additional variables were measured in the perinatal assessment and used in multivariate analyses to adjust for potential confounders: child's sex (male vs. female), maternal age in years (categorised as <20, 20–34, and ≥35), maternal education in completed school years (classified as 0–4, 5–8, 9–11, ≥12) and family income in quintiles (originally collected in minimum wages).

### Statistical analysis

A statistical analysis plan was pre‐registered on the OSF (available at https://osf.io/gwtfe/?view_only=ad5aee99a57a4e69b57e060ff04d2fc7). The analyses proceeded in five stages. First, we describe the sociodemographic characteristics of children included in the current study and for the entire cohort at baseline. Second, a cut‐off point to define children with higher CU traits was identified using ROC curve analyses (Table S1). Specifically, CU traits scores at age 4 were evaluated as a predictor of diagnosis of conduct disorder or oppositional defiant disorder when children were aged 6–7, and different possible cut‐points were examined for sensitivity and specificity. Using the cut‐point identified in this analysis, the sample was then split into four groups for the main analyses: (1) children lower in CU traits and lower in CP (typically developing), (2) children lower in CU traits and higher in CP (CP‐only), (3) children higher in CU traits and lower in CP (CU‐only) and (4) children higher in CU traits and higher in CP (CP + CU).

Third, we calculated descriptive statistics (% and means/standard deviations) for all 19 psychosocial characteristics, stratified by the four CU/CP groups. For count and continuous variables, ANOVA was used to compare the characteristics, and the chi‐squared test for dichotomous/categorical variables. Fourth, we used multivariate regression to estimate associations between the four CU/CP groups and all psychosocial characteristics, with the typically developing group as the reference. To test whether CU traits and CP had independent associations with other psychosocial characteristics, we conducted *post‐hoc* regression analyses, where CU traits and CP were entered as separate continuous variables into one regression model (for each outcome). Poisson regression was used for analyses of all psychosocial characteristics, except for impulse control, which was analysed using linear regression. From Poisson models, Incident Rate Ratios (IRR) were calculated for count variables, while prevalence ratios were estimated for dichotomous variables using robust variance (Barros & Hirakata, 2003). For the continuous variable of impulse control, beta coefficients were estimated. Associations are shown with 95% confidence intervals. Models were based on the sample with complete data on CU traits, CP, confounders and valid data on each psychosocial test (which differed slightly between tests). *p*‐values presented in all tables have been adjusted for multiple comparisons using the Benjamini‐Hochberg procedure. For all analyses, a significance level of 5% was used. All analyses were performed in STATA 15.1.

Exploratory analyses, that had not been pre‐registered, were conducted to better understand the observed relationships in the main analyses. Thus, we also examined each of CU traits and CP individually (in dichotomous form) regarding their relationship with psychosocial characteristics (i.e. comparing children higher vs. lower on CU traits in one set of analyses, and higher vs. lower in CP in another set of analyses; Table S2). Unadjusted and adjusted associations comparing higher versus lower CU trait groups with children's psychosocial characteristics are presented in Table S3. The same analyses were conducted for higher versus lower CP groups, as shown in Table S4. We also repeated the main analyses (examining four CU/CP groups) using more extreme cut‐off points to identify children with considerably higher levels of CU traits (≥14) and CP (≥6; Table S5), approximating to 10% of the sample for each (see Appendix S2 regarding these choice of cut‐points), as a form of sensitivity analyses considering the cut‐points used in the main analyses which identified larger numbers of children with higher CU traits and CP. Adjusted associations between four CU/CP groups, based on more extreme cut‐off points, and children's psychosocial characteristics are presented in Table S6. In addition, we repeated the main analyses stratifying the sample by sex, and tested interaction effects between CU/CP groups and sex to examine potential moderation (Table S7).

## Results

Of the 4,275 children assessed at birth, 3,997 were included in the main analyses of the current study, while 278 were excluded because of death (*n* = 67), loss to follow‐up at age 4 years (*n* = 198) and lack of CU traits or CP data (*n* = 13). Table 2 shows the sociodemographic characteristics of the analytic sample, which were very similar to the original cohort. Half (50.7%) of children in the analytic sample were male; most mothers were 20–34 years old (70.8%) and had nine or more years of schooling (65.2%).

**Table 2:** Baseline sociodemographic characteristics of the original cohort 2015 Pelotas Birth Cohort and of children included in the analytic sample

	Original cohort	Included in analyses
	*N* (%)	*N* (%)
Total	4,275	3,997
Sex		
Male	2,164 (50.62)	2,025 (50.66)
Female	2,111 (49.38)	1,972 (49.34)
Maternal age (years)		
<20	623 (14.58)	583 (14.59)
20–34	3,018 (70.61)	2,828 (70.77)
≥35	633 (14.81)	585 (14.64)
Maternal schooling (years)		
0–4	391 (9.15)	351 (8.78)
5–8	1,095 (25.62)	1,038 (25.98)
9–11	1,458 (34.11)	1,394 (34.88)
≥12	1,330 (31.12)	1,213 (30.36)
Family income		
1 (Lowest)	857 (20.06)	796 (19.92)
2	856 (20.03)	814 (20.38)
3	851 (19.92)	804 (20.13)
4	855 (20.01)	807 (20.20)
5 (Highest)	854 (19.99)	774 (19.37)

At age 4, the mean CU traits score for the total sample was 7.7 (SD = 4.4, range 0–31), with higher scores for boys (mean = 8.1, SD = 4.4) than girls (mean = 7.3, SD = 4.3; *p* < .001 for sex difference). Almost one third (31%, *n* = 1,239) of the sample (56.6% boys, 43.4% girls; *p* < .001 for sex difference) had elevated CU traits (based on a cut‐point of ≥10, identified in ROC analyses – see Appendix S2). The mean CP scores were 2.5 (SD = 2.0, range 0–10), with slightly higher average scores for boys (2.6; SD = 2.1, range 0–10) than girls (2.4; SD = 2.0, range 0–10; *p* < .001 for sex difference). Nearly one third of children (30.4%; *n* = 1,216) had elevated CP scores (54.2% boys, 45.8% girls; *p* = .003 for sex difference).

Four groups of children were classified jointly considering their levels of CU traits and CP. Over half of children (54.9%; *n* = 2,191) were classified as typically developing – without either higher CU traits or higher CP, 14.2% (*n* = 565) were classified as higher CP‐only, 14.8% (*n* = 589) as higher CU‐only and 16.3% (*n* = 649) higher CP + CU. Table 3 describes the psychosocial characteristics of these four CU/CP groups. There were significant group differences across all 19 measures, except for social information processing (three indicators) and one measure of altruism (Dictator Game). The typically developing group showed the best performance scores for all measures. Generally, the poorest test scores were observed among the two groups of children manifesting higher CU traits (CU‐only and CP + CU). The CP + CU group had the poorest performance scores on the communication domain of neurodevelopment, language, most of the executive functions scores, altruism and prosocial behaviour. Children with CU‐only had the poorest scores out of all four groups on several neurodevelopmental domains (social, adaptive, motor, cognitive), on emotion recognition, theory of mind, and empathy‐altruism (Help Task). Children with CP‐only had quite similar psychosocial characterisation as typically developing children on numerous tests but had somewhat poorer performance for gratification delay and attention shifting.

**Table 3:** Psychosocial characteristics of four groups of children with higher/lower callous‐unemotional (CU) traits and conduct problems (CP)

	Valid *N*	Range^a^	Typically developing	Higher	Higher	Higher	*p* Value
				CP‐only	CU‐only	CP and CU	
			*n* = 2,192	*n* = 566	*n* = 589	*n* = 650	
			*M* (*SD*) or %	*M* (*SD*) or %	*M* (*SD*) or %	*M* (*SD*) or %	
Neurodevelopment (Battelle scores)							
Social	3,827	4–28	25.62 (1.96)	25.19 (2.26)	24.48 (2.91)	24.51 (2.92)	.**005**
Adaptative	3,821	3–28	26.46 (1.71)	26.30 (1.95)	25.97 (2.58)	26.11 (2.03)	.**005**
Motor	3,750	10–28	23.10 (2.29)	22.95 (2.25)	22.50 (2.45)	22.80 (2.36)	.**005**
Communication	3,776	1–24	19.36 (2.47)	18.71 (2.46)	18.41 (3.11)	18.38 (2.81)	.**005**
Cognitive	3,783	0–24	20.28 (2.47)	19.63 (2.75)	19.22 (3.02)	19.25 (2.87)	.**005**
Language							
Expressive language	3,768	0–93	56.14 (19.27)	50.98 (19.69)	48.15 (20.12)	46.98 (20.76)	.**005**
Receptive language	3,835	0–33	25.78 (4.80)	24.41 (5.56)	23.54 (5.60)	23.09 (5.85)	.**005**
Executive functions							
Self‐control (interviewer rating)	3,893	4–12	10.21 (2.30)	9.46 (2.80)	9.61 (2.64)	9.15 (2.85)	.**005**
Gratification delay (Marshmallow task)	3,433	0–210	183.79 (59.30)	171.31 (71.35)	166.10 (75.15)	164.74 (73.17)	.**005**
Impulse control (EYT task)	2,854	0–1	0.36 (0.18)	0.34 (0.16)	0.33 (0.18)	0.33 (0.18)	.**005**
Attention shifting (EYT task)	3,806	0/1	15.6%	11.1%	11.2%	10.5%	.**005**
Emotion recognition/Theory of mind							
Emotion recognition	3,556	0–5	2.39 (1.05)	2.11 (1.07)	1.92 (1.07)	1.93 (1.06)	.**005**
Theory of Mind	3,778	0–3	1.74 (0.84)	1.65 (0.85)	1.60 (0.87)	1.68 (0.84)	.**005**
Social information processing							
Hostile attribution bias	3,229	0/1	71.4%	71.0%	66.5%	69.0%	.179
Aggressive response generation	2,969	0/1	11.9%	16.0%	12.9%	12.9%	.179
Competent assessment score	3,060	0–9	5.97 (1.92)	5.78 (1.91)	5.76 (1.93)	6.00 (1.87)	.179
Prosocial behaviour							
Empathy‐altruism (Help task)	3,706	0–3	1.31 (1.26)	1.27 (1.24)	1.09 (1.22)	1.13 (1.23)	.**005**
Altruism (Dictator game)	3,827	0–10	3.55 (2.66)	3.30 (2.67)	3.41 (2.79)	3.28 (2.63)	.179
Prosocial behaviour	3,997	0–10	9.22 (1.07)	8.87 (1.21)	8.16 (1.69)	7.60 (1.94)	.**005**

*Note*: Bold *p*‐values indicate statistical significance.

^a^ Range of observed values in the current sample. ANOVA for count and continuous variables. Chi‐squared test for dichotomous variables. *p*‐Values adjusted for multiple comparisons by Benjamini‐Hochberg procedure.

### Associations between CU traits, CPs and child psychosocial characteristics

Table 4 shows associations between CU/CP groups and psychosocial characteristics from regression models, with typically developing children as the reference category. Overall, these results highlight the poorer performance of children with higher CU traits, both when manifest alone (CU‐only) and combined with CPs (CP + CU). In fact, the magnitude of associations (ranges of IRRs summarised here) was very similar between children with CU‐only and children with both CP + CU: neurodevelopmental scores (0.96–0.98 CU‐only; 0.97–0.99 CP + CU); language scores (0.92–0.95 CU‐only; 0.92–0.94 CP + CU); executive functions (0.91–0.96 CU‐only; 0.91–0.92 CP + CU); emotion recognition/theory of mind (0.87–0.94 CU‐only; 0.89–0.99 CP + CU); and prosocial behaviour (0.86–0.98 CU‐only; 0.83–0.95 CP + CU). By contrast, many associations were null or considerably smaller for the CP‐only group. No significant associations were found for any of the CU/CP groups for eight variables: two measures of executive functions (impulse control and attention shifting), theory of mind, social information processing variables and two measures of altruism.

**Table 4:** Associations between groups of higher/lower callous‐unemotional (CU) traits and conduct problems (CP) and children's psychosocial characteristics

	Higher	Higher	Higher	*p*‐Value
	CP‐only	CU‐only	CP and CU	
	Reference category: Typically developing children			
	Adjusted IRR or *β* ^a^ (95% CI)			
Neurodevelopment (Battelle scores)				
Social	0.99 (0.98, 1.00)	**0.96 (0.95, 0.97)**	**0.97 (0.96, 0.98)**	.**010**
Adaptative	0.99 (0.99, 1.00)	**0.98 (0.97, 0.99)**	**0.99 (0.98, 0.99)**	.**010**
Motor	1.00 (0.99, 1.01)	**0.98 (0.97, 0.99)**	1.00 (0.99, 1.01)	.**036**
Communication	**0.98 (0.97, 0.99)**	**0.97 (0.95, 0.98)**	**0.97 (0.96, 0.98)**	.**010**
Cognitive	**0.98 (0.97, 0.99)**	**0.96 (0.95, 0.98)**	**0.97 (0.95, 0.98)**	.**010**
Language				
Expressive language	0.97 (0.93, 1.00)	**0.92 (0.89, 0.96)**	**0.92 (0.89, 0.95)**	.**010**
Receptive language	0.98 (0.96, 1.00)	**0.95 (0.93, 0.97)**	**0.94 (0.92, 0.96)**	.**010**
Executive Functions				
Self‐control (interviewer rating)	**0.94 (0.91, 0.96)**	**0.96 (0.93, 0.98)**	**0.92 (0.89, 0.94)**	.**010**
Gratification delay (Marshmallow task)	**0.94 (0.90, 0.98)**	**0.91 (0.88, 0.96)**	**0.91 (0.87, 0.95)**	.**010**
Impulse control (EYT task)^a^	−0.01 (−0.03, 0.01)	−0.02 (−0.04, 0.00)	−0.01 (−0.03, 0.01)	.483
Attention shifting (EYT task)^b^	0.84 (0.65, 1.09)	0.89 (0.69, 1.15)	0.89 (0.69, 1.15)	.483
Emotion recognition/Theory of mind				
Emotion Recognition	**0.94 (0.90, 0.99)**	**0.87 (0.83, 0.92)**	**0.89 (0.85, 0.94)**	.**010**
Theory of Mind	0.96 (0.91, 1.01)	**0.94 (0.89, 0.98)**	0.99 (0.95, 1.04)	.294
Social information processing				
Hostile attribution bias^b^	0.99 (0.93, 1.06)	**0.92 (0.86, 0.99)**	0.96 (0.90, 1.02)	.483
Aggressive response generation^b^	**1.33 (1.03, 1.72)**	1.07 (0.81, 1.41)	1.06 (0.80, 1.41)	.483
Competent assessment score	0.97 (0.94, 1.00)	0.97 (0.93, 1.00)	1.01 (0.97, 1.04)	.402
Prosocial behaviour				
Empathy‐altruism (Help task)	0.99 (0.90, 1.09)	**0.86 (0.77, 0.95)**	**0.90 (0.81, 0.99)**	.072
Altruism (Dictator game)	0.95 (0.88, 1.02)	0.98 (0.91, 1.06)	0.95 (0.88, 1.02)	.483
Prosocial behaviour	**0.96 (0.95, 0.98)**	**0.89 (0.87, 0.90)**	**0.83 (0.81, 0.84)**	.**010**

IRR, incidence rate ratio from Poisson regression (representing prevalence ratios for dichotomous variables^b^, based on robust error variance).

^a^ *β* from Linear Regression. Results shown in bold *p* < .05 or 95% CI does not include the null value. *p* Values adjusted for multiple comparisons by Benjamini‐Hochberg procedure. Adjusted for sex, maternal age, maternal schooling and family income.

To examine the extent to which CU traits and CP had independent associations with children's psychosocial characteristics, we conducted further analyses using continuous scores. Table 5 shows associations using standardised, continuous CU trait and CP scores (adjusted for each other as well as other covariates). CU traits were significantly associated with lower scores across all neurodevelopmental and language indicators, as well as lower self‐control, gratification delay, impulse control, emotion recognition, empathy‐altruism and prosocial behaviour. CP was associated only with lower self‐control and prosocial behaviour in these analyses. Neither CU traits nor CP were associated with attention shifting, hostile attribution bias, aggressive response generation, or competent assessment.

**Table 5:** Associations of callous‐unemotional (CU) traits and conduct problems (CP) with children's psychosocial characteristics, using continuous scores for CU traits and CP, adjusting for each other and additional covariates

	CU traits		CPs	
	Adjusted IRR or *β* ^a^ (95% CI)	*p*‐Value	Adjusted IRR or *β* ^a^ (95% CI)	*p*‐Value
Neurodevelopment (Battelle scores)				
Social	0.98 (0.98, 0.99)	.**008**	1.00 (0.99, 1.00)	.927
Adaptative	0.99 (0.99, 0.99)	.**008**	1.00 (1.00, 1.00)	.927
Motor	0.99 (0.99, 0.99)	.**008**	1.00 (1.00, 1.01)	.927
Communication	0.98 (0.97, 0.99)	.**008**	0.99 (0.99, 0.99)	.385
Cognitive	0.98 (0.98, 0.99)	.**008**	0.99 (0.99, 0.99)	.385
Language				
Expressive	0.96 (0.95, 0.98)	.**008**	0.98 (0.97, 0.99)	.165
Receptive	0.97 (0.97, 0.98)	.**008**	0.99 (0.98, 0.99)	.224
Executive functions				
Self‐control (interviewer rating)	0.98 (0.97, 0.99)	.**008**	0.97 (0.96, 0.98)	.**018**
Gratification delay (Marshmallow task)	0.97 (0.96, 0.99)	.**008**	0.98 (0.97, 0.99)	.136
Impulse control (EYT task)^a^	−0.01 (−0.02, −0.01)	.**014**	−0.00 (−0.01, 0.00)	.927
Attention shifting (EYT task)^b^	1.00 (0.91, 1.10)	.955	0.91 (0.82, 1.00)	.558
Emotion recognition/Theory of mind				
Emotion recognition	0.95 (0.93, 0.96)	.**008**	0.98 (0.96, 0.99)	.440
Theory of Mind	0.98 (0.97, 1.00)	.505	0.99 (0.98, 1.01)	.927
Social information processing				
Hostile attribution bias^b^	0.98 (0.95, 1.00)	.486	1.00 (0.97, 1.02)	.927
Aggressive response generation^b^	0.93 (0.83, 1.04)	.892	1.14 (1.03, 1.26)	.160
Competent assessment score	0.99 (0.98, 1.01)	.955	1.01 (0.99, 1.02)	.927
Prosocial behaviour				
Empathy‐altruism (Help Task)	0.93 (0.90, 0.97)	.**008**	1.00 (0.97, 1.04)	.927
Altruism (Dictator game)	0.99 (0.96, 1.02)	.955	0.97 (0.94, 0.99)	.385
Prosocial behaviour	0.93 (0.92, 0.93)	.**008**	0.98 (0.97, 0.99)	.**018**

IRR represents incidence rate ratio from Poisson regression (representing prevalence ratios for dichotomous variables^b^, based on robust error variance).

^a^ *β* from linear regression. *p*‐values adjusted for multiple comparisons by Benjamini‐Hochberg procedure. Adjusted for sex, maternal age, maternal schooling and family income.

### Exploratory analyses

Three further sets of results from exploratory analyses are detailed in Appendix S2 and summarised here. First, when CU traits and CP were examined separately, as dichotomous variables, children with higher vs. lower CU traits exhibited poorer performance across nearly all psychosocial measures, including all neurodevelopmental domains, language, self‐control, gratification delay, emotion recognition, hostile attribution bias and prosocial behaviour (Table S3). However, children with higher vs. lower CP showed deficits only in specific areas: the communication domain of neurodevelopment, receptive language, self‐control, gratification delay and one measure of prosocial behaviour (Table S4).

Second, in sensitivity analyses examining four CU/CP groups defined by more extreme cut‐points for higher CU traits (≥14) and higher CP (≥6), results were largely similar to the main analyses (based on a ≥10 cut‐off for CU traits and ≥4 for CP), except that associations with theory of mind and empathy‐altruism were no longer statistically significant (Table S6).

Results from a third set of exploratory analyses, stratifying by sex, are shown in Table S8. Although there were several interactions by sex, associations between the CU/CP groups and psychosocial characteristics were generally of similar magnitude for girls and boys. However, CU/CP group differences in the neurodevelopmental domains of adaptation and communication were evident in boys but not in girls (interaction by sex, *p* = .010).

## Discussion

In this large, population‐based cohort study in Brazil, we examined a broad range of psychosocial characteristics according to children's levels of CU traits and CPs. Although some previous studies of CU traits among pre‐schoolers have examined associated specific individual characteristics, this is the first attempt to provide a broader developmental profile of young children with higher CU traits. In the current study, CU traits were associated with meaningful differences across a wide range of child developmental indicators, including language and motor development, whereas child CPs on their own were mostly associated with factors directly related to disruptive behaviour, such as poor self‐control. Indeed, CU traits had stronger relationships with nearly all aspects of child psychosocial functioning compared to child CPs. These findings suggest a holistic understanding of young children manifesting CU traits is required with appropriate treatment models for associated CPs.

In this study, children with CPs only (without high CU traits) had few differences compared to typically developing children (without CPs or CU traits) on a broad range of psychosocial tests, suggesting that CPs in early childhood may not, on their own, reflect general developmental vulnerability. In contrast, children with higher CU traits, regardless of their level of CPs, displayed considerable challenges across a range of developmental domains, including motor development and language skills as well as other aspects such as low empathy, emotion recognition and self‐control, indicating that CU traits may signal more global neurodevelopmental risk than previously understood. Key theories highlight that neurodevelopmental difficulties are core features of early‐onset and persistent CPs (Moffitt, Lynam, & Silva, 1994), and the current findings suggest that this may principally reflect associations with CU traits.

An important finding in the current, population‐based study was the identification of a substantially sized group of children (14.75%) with elevated CU traits who were not manifesting high levels of CPs, reinforcing the importance of expanding research on CU traits beyond clinically referred children or other high‐risk samples. Notably, these children with higher CU traits but without high CPs demonstrated poorer theory of mind and lower hostile attribution bias compared to all other groups. This suggests that CU traits, in the absence of CPs, may reflect deeper impairment in understanding of others' mental states, along with a tendency to make more benign interpretations of others' intentions, and the importance of considering CU traits both with and independently of the manifestation of conduct behaviour.

While this study's finding on the significance of CU traits for children's wider developmental functioning certainly supports the use of a CU trait specifier for child CPs, it also points to considerably broader needs of children manifesting CU traits, both inside and outside the context of CP behaviour. The observed pattern of associations may help to identify key targets for early intervention, showing that CU traits are linked to poorer emotion recognition, empathy, theory of mind, language and broader neurodevelopmental functioning. These results align with evidence that CU traits reflect disruptions in emotional and socio‐cognitive processing (Blair et al., 2014; Waller et al., 2020). Taken together, the findings underscore the importance of early, developmentally informed interventions that prioritise emotional and social‐cognitive skills over an exclusive focus on behavioural symptom reduction (Northam & Dadds, 2020). Future research would benefit from complementary methodological approaches, including latent class analysis, to better capture heterogeneity within this population and examine whether additional subgroups emerge beyond our a priori categories, thereby informing more precise intervention strategies.

Although CU traits were uniquely associated with several aspects of child development in this study, other factors were linked to both CU traits and CPs. Consistent with other studies, poor executive functioning, particularly regarding self‐control and gratification delay, was associated with both CU traits and CPs (Graziano et al., 2019). This can be understood to reflect processes of self‐regulation and frustration that are directly involved in CP behaviours, rather than broader developmental processes, which related more strongly to CU traits.

It is important to clarify how these findings should be interpreted given the large sample, and probability of statistical significance even when effect sizes are small. Consistent with this, most individual associations, even when significant, were modest in magnitude. As such, instead of focussing on individual coefficients, we emphasise the direction and consistency of effects observed. Even with modest effect sizes, the fact that findings were directionally consistent across a wide domain of measures may have meaningful implications about developmental patterns, particularly in relation to CU traits.

Another contribution of the current study providing data on potential cut‐points for the Inventory of Callous‐Unemotional traits (ICU), towards identifying young children at risk for disruptive behaviour disorders. Given the widespread use of the ICU in research settings (Bedford et al., 2021; Hartmann & Schwenck, 2020; Muñoz, 2009), efforts to establish optimal cut‐points are essential for improving identification and early intervention strategies. Although the ROC curve analysis in the current study did not clearly indicate a unique optimal cut‐off, a mathematical approach allowed us to define a threshold that best balanced sensitivity and specificity. Notably, a higher alternative cut‐off point (≥14), which was more specific, yielded similar associations with psychosocial characteristics.

This study had important strengths in terms of its population‐based sample of young children, a wide range of developmental assessments made directly with children as well as their caregivers, and longitudinal data that supported identification of levels of CU traits most indicative of later behaviour disorders. However, findings should be interpreted in the light of study limitations. The key limitation of this study is that differential sensitivity of the two main measures used to measure CU traits and CPs could have contributed to one (CU traits) appearing a more important construct than the other. Notably, the ICU used to measure CU traits consists of 12 items, while the SDQ sub‐scale on CPs has five. Both are well‐established measures, but potentially a more extensive instrument to evaluate CPs could have revealed stronger associations with other study variables. There are other more generic limitations to other measures used: parent‐reported questionnaires can be influenced by subjective perceptions, social desirability and caregiver emotional states, while children's task performance may be affected by motivation, fatigue, attention span or unfamiliarity with the testing context. Another limitation is that analyses were not adjusted for other psychiatric or neurodevelopmental diagnoses, potentially resulting in uneven comorbidity across CU/CP profiles; however, this was intentional to examine dimensional associations independent of diagnostic status. Lastly, analyses were adjusted using information on mothers only, due to high levels of missing data on paternal characteristics and strong collinearity between maternal and paternal sociodemographic indicators.

In conclusion, this study suggests that children with elevated CU traits show impairments across broad developmental domains, whereas CPs are associated with more specific emotional and self‐control difficulties. These findings underscore the need for early, developmentally informed support addressing diverse neurodevelopmental and psychosocial needs, particularly among children with higher CU traits.

## Ethical considerations

Informed parental consent/assent has been appropriately obtained for all interviews, and participants were free to stop assessments at any time. The study assessments referring to data collection from the perinatal period through 4 years of age were approved on 5 February 2014, by the Ethics Committee of the Physical Education School, Federal University of Pelotas (protocol number 26746414.5.0000.5313). Additionally, ethical approval was obtained from the Ethics Committee of the Medical School, Federal University of Pelotas (protocol number 51789921.1.0000.5317), for the follow‐up assessments conducted at 6–7 years of age. Approval was granted on 12 October 2021.

**Key points**

**What is known?**

- Callous‐unemotional (CU) traits and conduct problems in childhood are well‐established predictors of chronic antisocial behaviour and other adverse outcomes.
- CU traits in pre‐schoolers are associated with widespread developmental difficulties, including language, neurodevelopment and prosocial behaviour challenges.

**What is new?**

- While CU traits are linked to broad developmental delays, conduct problems on their own are mainly deficits in self‐control and emotion regulation, not broader developmental delays.

**What is relevant?**

- Findings emphasise the importance of a holistic approach to understanding and treating young children with higher levels of CU traits.

## Supporting information


**Appendix S1.** Details of psychological assessments.
**Appendix S2.** ROC curve analyses.
**Table S1.** Sensitivity, specificity, distance and balance for each potential cut‐point in the ROC curve analyses.
**Table S2.** Descriptive statistics of psychosocial characteristics by levels of callous unemotional (CU) traits and, separately, by levels of conduct problems.
**Table S3.** Associations between higher/lower callous unemotional (CU) traits groups and children's psychosocial characteristics, adjusting for conduct problems and additional covariates.
**Table S4.** Associations between higher/lower conduct problems groups and children's psychosocial characteristics, adjusting for callous unemotional (CU) traits and additional covariates.
**Table S5.** Psychosocial characteristics of four groups of children higher/lower in callous unemotional (CU) traits (cut‐off ≥ 14) and conduct problems (cut‐off ≥ 6).
**Table S6.** Associations between groups of higher/lower callous unemotional traits (cut‐off ≥ 14) and conduct problems (cut‐off ≥ 6) and children's psychosocial characteristics, adjusting for covariates.
**Table S7.** Psychosocial characteristics of children with higher/lower callous unemotional traits and conduct problems, stratified by sex.
**Table S8.** Associations between groups of higher/lower callous unemotional traits and conduct problems and children's psychosocial characteristics stratifying by sex and adjusting for covariates and *p*‐value of interaction term between group and sex.

## Data Availability

L.R.P. had full access to all the data in the study and takes responsibility for the integrity of the data and the accuracy of the data analysis. Collaborative use of 2015 Pelotas Birth Cohort Study data is available to the Study coordinating committee upon request from the corresponding author. The data are not publicly available due to privacy or ethical restrictions.
