# Supplementary material for: Conduct problems and callous‐unemotional traits in pre‐schoolers: Psychosocial profiles in a Brazilian birth cohort
Source: J Child Psychol Psychiatry. 2026 May 16;67(10):1639–48. doi: 10.1111/jcpp.70164 (PMC13535731; doi:10.1111/jcpp.70164)
Supplement: Supplementary file 1 [file JCPP-67-1639-s001.docx]

**Conduct Problems and Callous-Unemotional Traits in Preschoolers: Psychosocial Profiles in a Brazilian Birth Cohort**

**Supporting Information**

[Appendix S1. DETAILS OF PSYCHOLOGICAL ASSESSMENTS	2](#_Toc201587560)

[Neuropsychomotor Development	2](#_Toc201587561)

[Language	2](#_Toc201587562)

[Executive functions	2](#_Toc201587563)

[Emotion recognition/Theory of mind	3](#_Toc201587564)

[Social Information Processing (SIP)	4](#_Toc201587565)

[Prosocial behaviour	4](#_Toc201587566)

Appendix S2. [ROC CURVE ANALYSES	5](#_Toc201587567)

[Table S1. Sensitivity, specificity, distance, and balance for each potential cut point in the ROC curve analyses.	5](#_Toc201587568)

[Table S2. Descriptive statistics of psychosocial characteristics by levels of callous unemotional (CU) traits and, separately, by levels of conduct problems (CP).	7](#_Toc201587569)

[Table S3. Associations between higher/lower callous unemotional (CU) traits groups and children's psychosocial characteristics, adjusting for conduct problems (CP) and additional covariates.	8](#_Toc201587570)

[Table S4. Associations between higher/lower conduct problems (CP) groups and children's psychosocial characteristics, adjusting for callous unemotional (CU) traits and additional covariates.	9](#_Toc201587571)

[Table S5. Psychosocial characteristics of four groups of children higher/lower in callous unemotional (CU) traits (cut-off ≥14) and conduct problems (CP) (cut-off ≥6).	10](#_Toc201587572)

[Table S6. Associations between groups of higher/lower callous unemotional (CU) traits (cut-off ≥14) and conduct problems (CP) (cut-off ≥6) and children's psychosocial characteristics, adjusting for covariates.	11](#_Toc201587573)

[Table S7. Psychosocial characteristics of children with higher/lower callous unemotional (CU) traits and conduct problems (CP), stratified by sex.	12](#_Toc201587574)

[Table S8. Associations between groups of higher/lower callous unemotional (CU) traits and conduct problems (CP) and children’s psychosocial characteristics stratifying by sex and adjusting for covariates, and *p* value of interaction term between group and sex.	13](#_Toc201587575)

[REFERENCES	14](#_Toc201587576)

Appendix S1. DETAILS OF PSYCHOLOGICAL ASSESSMENTS

Neuropsychomotor Development

Neurospsychomotor development was measured by the Battelle’s Development Inventory – screening version (BDI) (Barros et al., 2010). This is a standardized tool used to evaluate child development in five domains: 1) personal-social, 2) adaptative, 3) fine and gross motor, 4) communication, and 5) cognitive. The BDI is completed by parents, applied directly to children, and observed by examiners. Sixty-six items were used, each rated on a three-point scale (0 = rarely or never, 1 = sometimes, 2 = often). We used the domains (i.e., personal-social, adaptative, motor, communicative, and cognitive) separately. The first three domains had a scale ranging from 0 to 28, while the last two ranged from 0 to 24. The higher the score, the higher the development in each domain.

Language

Expressive language was assessed by the University of São Paulo (USP) Expressive Vocabulary Test (TVexp-100), which is a picture-naming test where the interviewer shows a picture book to the child and asks them to name the pictures, i.e., “What is the name of…”. This instrument is composed of 100 items applied directly to the child. Each item is dichotomous (scored 1 for a correct answer and 0 for incorrect), and all are summed to compute the total score of the measure (ranging from 0 to 100). Receptive language was assessed using the USP Auditory Vocabulary Test (TVAud-33), composed of 33 items (Capovilla et al., 2011). Each item shows five pictures in a line, and the interviewer says a word to the child, who is asked to point to the corresponding picture. When the child successfully finds the picture, one point is added to the total score (ranging from 0 to 33). In this study, the language variable was scored by the sum of the two aforementioned scores and ranged from 0 to 133. The higher the score, the higher the language development.

Executive functions

The executive functions considered in this paper were self-control, gratification delay, impulse control, and attention shifting. Self-control was reported based on the interviewer’s impressions of the child’s behaviour while completing the other cognitive and motor tasks at 4 years follow-up (Caspi et al., 1995). The measure comprises four items about fleeting attention, negativism, restlessness, and emotional lability presented by the child during the tests. Behavioural characteristics are rated on three scale points, with the high end indicating a higher level of that attribute. The final range goes from 4 to 12.

The gratification delay measure was given by a task in which two plates of candies were placed on the table in front of the child, one with one candy and the other with three. Children were told they could eat from the plate with only one candy any time they wanted after the interviewer left the room. Still, if they wished to have a large plate with more (3) candies, they would have to wait and eat nothing until the interviewer returned (after 3 minutes and 30 seconds). In the procedure, the interviewer then leaves and observes the child through the “magic eye” of the door and counts the time in seconds until the child eats the candy. The delay of gratification is then recorded as the number of seconds the child waited, with 3 minutes and 30 seconds being the ceiling. The final range goes from 0 to 210.

The impulse control was measured by the Early Years Toolbox Go/No-Go (Howard & Melhuish, 2017). This task requires participants to tap the screen for ‘go’ trials (‘catch the fish’) and not tap the screen for ‘no-go’ trials (‘avoid catching sharks’). Most stimuli were ‘go’ trials (80% fish), which generated a pre-potent tendency to respond, requiring participants to inhibit this response on no-go trials (20% sharks). Before commencing, participants were given instructions and practice as follows: go instructions, followed by five practice ‘go’ trials; no-go instructions, followed by five practice ‘no-go’ trials; combined go/no-go instructions, followed by a mixed block of 10 practice trials (80% go trials); and a recap of instructions. The task proceeds with 75 stimuli presented in pseudo-random order. Each trial involves the presentation of an animated stimulus (i.e., fish or shark). The final score, which continuously ranges from 0 to 1, is the product of proportional ‘go’ (for the strength of the pre-potent response generated) and ‘no-go’ accuracy (to index a participant’s ability to overcome this pre-potent response). Higher scores indicate better impulse control.

The attention shifting was assessed by the Early Years Toolbox Card Sorting (Zelazo, 2006). The Card Sorting Task is an iPad-based assessment in which children are presented with cards that vary along two dimensions (i.e., shape and colour) and asked to sort each card (i.e., red rabbits and blue boats) first by one dimension (e.g., colour), and after some trials, by another dimension (e.g., shape). If the participant correctly sorts these first two blocks (pre- and post-switch stimuli), they proceed to a border phase of the task (3^rd^ block). During this phase, children are tasked with sorting based on colour and shape, assessing their ability to shift between complex criteria. Their ability to flexibly shift from one sorting rule to another corresponds to their cognitive flexibility (or 'shifting'). For this paper, we dichotomized this variable in 0 = child who could not reliably simply shift (i.e., poorly completed block 1 and block 2) and 1 = child who could reliably simply shift (i.e., satisfactorily completed block 2).

Emotion recognition/Theory of mind

For the emotion recognition measure, photos of children’s faces showing different emotional expressions (portraying happiness, sadness, anger, and fear) are shown to the child on puppets, and the child is asked to identify the emotions by naming and pointing. The task is divided into two parts: first, children are asked four questions, such as "What is this puppet feeling?" to assess expressive knowledge. Second, children are shown four puppets with different emotional expressions and asked to point to the puppet that represents a specific emotion, for example, "Which puppet feels happy?" to assess receptive knowledge. The child receives a score of 2 for correctly naming the emotion or identifying the correct emotion, a score of 1 for a response that correctly reflects the emotion's valence or direction but is not entirely accurate, and a score of 0 is assigned when neither the correct emotion nor the correct valence/direction is identified. The overall emotion recognition score is calculated as the mean of the expressive and receptive knowledge scores, ranging from 0 to 8.

The Theory of Mind Understanding (TOM) was measured by the Sally-Anne test (Fujino et al., 2017). This instrument is a ‘story task,’ with two-story characters invoked concerning a hidden object. In the procedure, Sally (Sophia in Pelotas) places a ball in a basket and leaves the room. Another character, Anne (called Ana in Pelotas), comes in and moves the ball to a box. As Sally returned, the children were asked, ‘Where will Sally look for her ball?’ (the *belief* question). If children understand that Sally’s actions will be based on her false belief (that the ball is where she had left it) rather than the actual location (that the ball is in Anne’s box), they should respond that she will look in the basket and thus answer the question correctly. If children pointed to the ball’s current location, they answered the question incorrectly and failed the test. Two other questions, ‘Where is the ball really?’ (the *reality* question) and ‘Where was the ball in the beginning?’ (the *memory* question) were also made. Children receive one point for each correct answer of the three items, so the final score ranges from 0 to 3.

Social Information Processing (SIP)

The SIP was measured by The Social Information Processing Interview – Preschool Version (SIPI-P), a structured interview that depicted a vignette in which two other peers reject the protagonist. The peers’ intent is portrayed as ambiguous. The interviewer told the story using a storybook easel with illustrations of bears. As the children hear the story, the interviewer stops at scripted points and poses questions addressing the hypothesized information-processing steps. After presenting the vignette, the interviewer asks the child whether the other children are mean or not mean. Children who answered that the other children were mean were categorized as having hostile attribution bias. Next, the interviewer asks an open-ended question: “What would you say or do if this happened to you?’’. These answers were dichotomized as aggressive responses or not. After that, the interviewer presents possible competent (e.g., asking the other children if he can play next), aggressive (e.g., kicking the blocks), and inept (e.g., crying) responses and asks questions about possible outcomes of such responses, creating a score that ranges from 0 to 9 (Ziv & Sorongon, 2011).

Prosocial behaviour

Three tests were used to measure prosocial behaviour. The first one, the Help Task, consists of a filmed task. The examiner feigns experiencing a problem (e.g., being unable to find something, such as a glue stick or a pen, that is in the child's view), and the child’s response is observed (Buttelmann et al., 2009). Trained undergraduate psychologists did the coding work under the supervision of a senior psychologist. Scores range from 0 to 3, with 3 being the highest helping behaviour (the child picks up the glue stick and returns it to the experimenter – it is clear that the child intends to return the object to the experimenter). Because of problems with experimenter compliance, an additional “0.5” score was created to indicate cases where the child picked up the glue stick and retained it under conditions where the experimenter had not clearly indicated their need to receive the glue stick back.

The Dictator Game was the second test used to measure prosocial behaviour, specifically altruism (Benenson et al., 2007). In this task, the experimenter showed the child a set of 10 stickers, saying ‘‘these stickers are yours to keep’’. But after a while, she explained that the stickers were finished and there wouldn’t be enough for another child, so not everyone could receive 10 stickers. The child was informed that if he/she wants to donate some stickers to another girl/boy in the clinic, the child should place the stickers in a box. The child should place the stickers that the child wanted to keep for himself in another box. After approximately 2 min, the number of stickers in each box was counted. The child's final score reflects the number of sticks donated, ranging from 0 to 10.

The third measure of prosocial behaviour was assessed by the parent-report Strengths and Difficulties Questionnaire (SDQ) prosocial subscale, which asks about child’s prosocial behaviour in the past 6 months (Goodman, 1999). It consists of five questions that are scored on a three-point Likert scale (0 = “false,” 1 = “more or less true,” and 2 = “true”), with a final range of 0 to 10. Higher scores reflect higher prosocial behaviour.

Appendix S2. ROC CURVE ANALYSES

We conducted ROC curve analyses to determine the optimal cut-point for categorizing children into higher and lower levels of callous-unemotional (CU) traits. Specifically, we evaluated CU traits scores at age 4 years as predictors of diagnosis of conduct disorder and/or oppositional defiant disorder when children were aged 6-7 years, measured with the DAWBA and based on ICD-10 criteria. For each potential cut-point, we first calculated the distance to the upper-left diagonal of the ROC curve using the formula: *d = sqrt((1-SENSITIVITY)^2 +(1-SPECIFICITY)^2)*. We also evaluated the balance between specificity and sensitivity using Younden's J statistic: *J = SENSITIVITY + SPECIFICITY - 1*. The cut-point that minimized the distance and provided a good balance between sensitivity and specificity was identified as ≥10 (Table S1).

| Table S1. Sensitivity, specificity, distance, and balance for each potential cut point in the ROC curve analyses. | | | | |
| --- | --- | --- | --- | --- |
| **Cutoff point** | **Sensitivity** | **Specificity** | **Distance** | **Balance** |
| ≥0 | 1 | 0 | 1 | 0 |
| ≥1 | 1 | 0,023 | 0,977 | 0,023 |
| ≥2 | 0,9697 | 0,0544 | 0,946085329 | 0,0241 |
| ≥3 | 0,9697 | 0,0982 | 0,902308888 | 0,0679 |
| ≥4 | 0,9091 | 0,166 | 0,838939098 | 0,0751 |
| ≥5 | 0,8485 | 0,2416 | 0,773383999 | 0,0901 |
| ≥6 | 0,8182 | 0,3225 | 0,701468096 | 0,1407 |
| ≥7 | 0,7879 | 0,4156 | 0,621699099 | 0,2035 |
| ≥8 | 0,7879 | 0,5157 | 0,52870871 | 0,3036 |
| ≥9 | 0,6364 | 0,6008 | 0,539968147 | 0,2372 |
| **≥10** | **0,6061** | **0,6775** | **0,509080996** | **0,2836** |
| ≥11 | 0,5152 | 0,7454 | 0,547587619 | 0,2606 |
| ≥12 | 0,4545 | 0,7979 | 0,581734183 | 0,2524 |
| ≥13 | 0,4242 | 0,8501 | 0,594992143 | 0,2743 |
| ≥14 | 0,3636 | 0,8911 | 0,645650192 | 0,2547 |
| ≥15 | 0,303 | 0,9225 | 0,701295409 | 0,2255 |
| ≥16 | 0,1818 | 0,9471 | 0,819908318 | 0,1289 |
| ≥17 | 0,1515 | 0,9609 | 0,849400412 | 0,1124 |
| ≥18 | 0,1212 | 0,977 | 0,879100927 | 0,0982 |
| ≥19 | 0,1212 | 0,9831 | 0,878962485 | 0,1043 |
| ≥20 | 0,0606 | 0,9896 | 0,939457567 | 0,0502 |
| ≥21 | 0,0606 | 0,9939 | 0,939419805 | 0,0545 |
| ≥22 | 0,0303 | 0,9958 | 0,969709096 | 0,0261 |
| ≥23 | 0,0303 | 0,9965 | 0,969706316 | 0,0268 |
| ≥24 | 0 | 0,9981 | 1,000001805 | -0,0019 |
| ≥25 | 0 | 0,9988 | 1,00000072 | -0,0012 |
| ≥27 | 0 | 0,9996 | 1,00000008 | -0,0004 |
| >27 | 0 | 1 | 1 | 0 |

**EXPLORATORY ANALYSES**

We conducted three sets of additional exploratory analyses (that were not pre-registered) to document the associations between callous-unemotional (CU) traits and conduct problems (CP) and child psychosocial characteristics considering alternative analytic procedures.

First, we examined each of CU traits and CP individually (in dichotomous form) regarding their relationship with psychosocial characteristics. In these analyses we used two separate variables: higher/lower CU traits, and higher/lower CP rather than the four-group variable used in the main analysis. Thus, each of higher/lower CU traits, and higher/lower CP, was examined in relation to child psychosocial characteristics separately. For each, descriptive data are shown in Table S2. Tables S3 and S4 show their associations with children’s psychometric characteristics.

Second, we repeated the main analyses, again using four CU/CP groupings, but this time defining children with higher CU traits and CP using higher cut-points on each scale. For CU traits, a cutoff of ≥14 (instead of ≥10) was used, which identified 10.4% of the sample as having higher CU traits, similar to the study by Viding et al. (2005). For CP, we used the instrument cut-off of ≥6 (instead of ≥4) for very high conduct problems (Goodman, 1999), resulting in 8.3% of the sample with higher CP. Based on these alternative (more specific) cut-off points, descriptive analyses are shown in Table S5, and associations with child psychosocial characteristics are shown in Table S6.

Third, we repeated the main analyses again, but separately for males and females, and tested the interaction between CU/CP groups and sex for each psychosocial characteristic. Descriptive results are shown in Table S7 and associations and interaction *p* values in Table S8.

Table S2. Descriptive statistics of psychosocial characteristics by levels of callous unemotional (CU) traits and, separately, by levels of conduct problems (CP).

|  | **Lower CU traits** | **Higher CU traits** |  | **Lower CP** | **Higher CP** |  |
| --- | --- | --- | --- | --- | --- | --- |
|  | n = 2,758  (69.0%) | n = 1,239 (31.0%) |  | n = 2,782 (69.6%) | n = 1,216 (30.4%) |  |
|  | **M (SD) or %** | **M (SD) or %** | ***p* value** | **M (SD) or %** | **M (SD) or %** | ***p* value** |
| **Neurodevelopment (Battelle scores)** |  |  |  |  |  |  |
| Social | 25.53 (2.03) | 24.50 (2.91) | **0.006** | 25.39 (2.24) | 24.83 (1.93) | **0.010** |
| Adaptative | 26.43 (1.77) | 26.04 (2.30) | **0.006** | 26.36 (1.93) | 26.20 (1.99) | 0.112 |
| Motor | 23.07 (2.28) | 22.66 (2.40) | **0.006** | 22.98 (2.33) | 22.87 (2.31) | 0.609 |
| Communication | 19.22 (2.48) | 18.40 (2.95) | **0.006** | 19.16 (2.64) | 18.53 (2.66) | **0.010** |
| Cognitive | 20.15 (2.54) | 19.24 (2.94) | **0.006** | 20.06 (2.63) | 19.43 (2.82) | **0.010** |
| **Language** |  |  |  |  |  |  |
| Expressive | 55.08 (19.46) | 47.53 (20.46) | **0.006** | 54.51 (19.71) | 48.86 (20.36) | **0.010** |
| Receptive | 25.50 (5.00) | 23.30 (5.74) | **0.006** | 25.50 (5.00) | 23.30 (5.74) | **0.010** |
| **Executive functions** |  |  |  |  |  |  |
| Self-control (interviewer rating) | 10.06 (2.43) | 9.37 (2.76) | **0.006** | 10.09 (2.39) | 9.29 (2.83) | **0.010** |
| Gratification delay (Marshmallow task) | 181.27 (62.10) | 165.38 (74.07) | **0.006** | 180.30 (63.11) | 167.85 (72.35) | **0.010** |
| Impulse control (EYT task) | 0.36 (0.18) | 0.33 (0.18) | **0.006** | 0.36 (0.18) | 0.33 (0.17) | **0.027** |
| Attention Shifting (EYT task) | 14.7% | 10.8% | **0.006** | 14.70% | 10.8% | **0.010** |
| **Emotion recognition/Theory of mind** |  |  |  |  |  |  |
| Emotion recognition | 2.33 (1.06) | 1.93 (1.06) | **0.006** | 2.29 (1.07) | 2.02 (1.07) | **0.010** |
| Theory of Mind | 1.73 (0.84) | 1.64 (0.85) | **0.030** | 1.71 (0.85) | 1.67 (0.84) | 0.550 |
| **Social information processing** |  |  |  |  |  |  |
| Hostile attribution bias | 71.4% | 67.8% | 0.168 | 70.4% | 69.9% | 0.786 |
| Aggressive response generation | 12.8% | 12.9% | 0.901 | 12.1% | 14.4% | 0.546 |
| Competent assessment score | 5.93 (1.92) | 5.89 (1.90) | 0.901 | 5.93 (1.93) | 5.90 (1.89) | 0.786 |
| **Prosocial behaviour** |  |  |  |  |  |  |
| Empathy-altruism (Help Task) | 1.30 (1.25) | 1.11 (1.22) | **0.006** | 1.26 (1.25) | 1.20 (1.23) | 0.609 |
| Altruism (Dictator game) | 3.50 (2.66) | 3.34 (2.70) | 0.276 | 3.52 (2.69) | 3.29 (2.65) | 0.112 |
| Prosocial behaviour | 9.15 (1.11) | 7.87 (1.85) | **0.006** | 9.15 (1.11) | 7.87 (1.85) | **0.010** |

***Note.*** Student T test for count and continuous variables. Chi-square test for dichotomous variables. *p* values adjusted for multiple comparisons by Benjamini-Hochberg procedure.

Table S3. Associations between higher/lower callous unemotional (CU) traits groups and children's psychosocial characteristics, adjusting for conduct problems (CP) and additional covariates.

|  | **Higher versus Lower CU traits** |  | **Higher versus Lower CU traits** |  | |
| --- | --- | --- | --- | --- | --- |
|  | **Unadjusted IRR or β* (95% CI)** | ***p* value** | **Adjusted IRR or β* (95% CI)** | ***p* value** | |
| **Neurodevelopment (Battelle scores)** |  |  |  |  | |
| Social | 0.96 (0.95, 0.97) | **0.006** | 0.97 (0.96, 0.98) | **0.009** | |
| Adaptative | 0.99 (0.98, 0.99) | **0.006** | 0.99 (0.98, 0.99) | **0.009** | |
| Motor | 0.98 (0.98, 0.99) | **0.006** | 0.99 (0.98, 0.99) | **0.016** | |
| Communication | 0.96 (0.95, 0.97) | **0.006** | 0.98 (0.97, 0.99) | **0.009** | |
| Cognitive | 0.95 (0.95, 0.96) | **0.006** | 0.97 (0.96, 0.98) | **0.009** | |
| **Language** |  |  |  |  | |
| Expressive | 0.86 (0.84, 0.89) | **0.006** | 0.93 (0.91, 0.96) | **0.009** | |
| Receptive | 0.91 (0.90, 0.93) | **0.006** | 0.96 (0.94, 0.97) | **0.009** | |
| **Executive Functions** |  |  |  |  | |
| Self-control (interviewer rating) | 0.93 (0.91, 0.95) | **0.006** | 0.96 (0.95, 0.98) | **0.009** | |
| Gratification delay (Marshmallow task) | 0.91 (0.88, 0.94) | **0.006** | 0.93 (0.90, 0.97) | **0.009** | |
| Impulse control (EYT task)* | -0.03 (-0.04, -0.01) | **0.006** | -0.01 (-0.03, 0.00) | 0.766 | |
| Attention Shifting (EYT task)† | 0.74 (0.61, 0.89) | **0.006** | 0.95 (0.78, 1.16) | 0.766 | |
| **Emotion recognition/Theory of mind** |  |  |  |  | |
| Emotion Recognition | 0.83 (0.80, 0.86) | **0.006** | 0.90 (0.87, 0.94) | **0.009** | |
| Theory of Mind | 0.95 (0.92, 0.99) | **0.030** | 0.97 (0.94, 1.01) | 0.766 | |
| **Social Information Processing** |  |  |  |  | |
| Hostile attribution bias† | 0.95 (0.90, 0.99) | 0.188 | 0.94 (0.89, 0.99) | 0.224 | |
| Aggressive response generation† | 1.01 (0.83, 1.24) | 0.901 | 0.94 (0.75, 1.18) | 0.766 | |
| Competent assessment score | 0.99 (0.97, 1.02) | 0.901 | 1.00 (0.97, 1.02) | 0.766 | |
| **Prosocial behaviour** |  |  |  |  | |
| Empathy-altruism (Help Task) | 0.86 (0.79, 0.92) | **0.006** | 0.88 (0.81, 0.95) | **0.009** | |
| Altruism (Dictator game) | 0.95 (0.90, 1.01) | 0.291 | 0.99 (0.93, 1.05) | 0.766 | |
| Prosocial behaviour | 0.86 (0.85, 0.87) | **0.006** | 0.87 (0.86, 0.89) | **0.009** | |
| ***Note.*** IRR: Incidence Rate Ratio from Poisson Regression (representing Prevalence Ratios for dichotomous variables†, based on robust error variance). *β from Linear Regression. *p* values adjusted for multiple comparisons by Benjamini-Hochberg procedure. Adjusted for sex, maternal age, maternal schooling and family income. | | | | |  |

Table S4. Associations between higher/lower conduct problems (CP) groups and children's psychosocial characteristics, adjusting for callous unemotional (CU) traits and additional covariates.

|  | **Higher versus Lower CP** |  | **Higher versus Lower CP** |  |
| --- | --- | --- | --- | --- |
|  | **Unadjusted IRR or β* (95% CI)** | ***p* value** | **Adjusted IRR or β* (95% CI)** | ***p* value** |
| **Neurodevelopment (Battelle scores)** |  |  |  |  |
| Social | 0.98 (0.97, 0.98) | **0.010** | 1.00 (0.99, 1.00) | 0.913 |
| Adaptative | 0.99 (0.99, 0.99) | 0.119 | 1.00 (0.99, 1.00) | 0.913 |
| Motor | 1.00 (0.99, 1.00) | 0.606 | 1.00 (1.00, 1.01) | 0.913 |
| Communication | 0.97 (0.96, 0.98) | **0.010** | 0.99 (0.98, 0.99) | 0.495 |
| Cognitive | 0.97 (0.96, 0.98) | **0.010** | 0.99 (0.98, 1.00) | 0.913 |
| **Language** |  |  |  |  |
| Expressive | 0.90 (0.87, 0.92) | **0.010** | 0.98 (0.95, 1.00) | 0.913 |
| Receptive | 0.94 (0.92, 0.95) | **0.010** | 0.98 (0.97, 0.99) | 0.448 |
| **Executive Functions** |  |  |  |  |
| Self-control (interviewer rating) | 0.92 (0.90, 0.94) | **0.010** | 0.95 (0.93, 0.96) | **0.018** |
| Gratification delay (Marshmallow task) | 0.93 (0.90, 0.96) | **0.010** | 0.96 (0.93, 0.99) | 0.221 |
| Impulse control (EYT task)* | -0.02 (-0.04, -0.01) | **0.027** | -0.01 (-0.02, 0.01) | 0.913 |
| Attention Shifting (EYT task)† | 0.73 (0.61, 0.89) | **0.010** | 0.90 (0.74, 1.10) | 0.913 |
| **Emotion recognition/Theory of mind** |  |  |  |  |
| Emotion Recognition | 0.88 (0.85, 0.91) | **0.010** | 0.97 (0.94, 1.01) | 0.913 |
| Theory of Mind | 0.97 (0.94, 1.01) | 0.560 | 1.00 (0.96, 1.04) | 0.913 |
| **Social Information Processing** |  |  |  |  |
| Hostile attribution bias† | 0.99 (0.95, 1.04) | 0.787 | 1.01 (0.96, 1.06) | 0.913 |
| Aggressive response generation† | 1.19 (0.97, 1.45) | 0.540 | 1.19 (0.96, 1.48) | 0.913 |
| Competent assessment score | 0.99 (0.97, 1.02) | 0.787 | 1.00 (0.97, 1.02) | 0.913 |
| **Prosocial behaviour** |  |  |  |  |
| Empathy-altruism (Help Task) | 0.95 (0.88, 1.02) | 0.606 | 1.01 (0.94, 1.09) | 0.913 |
| Altruism (Dictator game) | 0.93 (0.89, 0.99) | 0.119 | 0.96 (0.90, 1.01) | 0.913 |
| Prosocial behaviour | 0.91 (0.90, 0.92) | **0.010** | 0.95 (0.94, 0.96) | **0.018** |
| ***Note.*** IRR: Incidence Rate Ratio from Poisson Regression (representing Prevalence Ratios for dichotomous variables†, based on robust error variance). *β from Linear Regression. *p* values adjusted for multiple comparisons by Benjamini-Hochberg procedure. Adjusted for sex, maternal age, maternal schooling and family income. | | | | |

Table S5. Psychosocial characteristics of four groups of children higher/lower in callous unemotional (CU) traits (cut-off ≥14) and conduct problems (CP) (cut-off ≥6).

|  |  |  | **Typically developing** | **Higher**  **CP-only** | **Higher**  **CU-only** | **Higher**  **CP+CU** |  |
| --- | --- | --- | --- | --- | --- | --- | --- |
|  |  |  | n = 2,660 | n = 922 | n = 121 | n = 294 |  |
|  | **Valid N** | **Range*** | **M (SD) or %** | **M (SD) or %** | **M (SD) or %** | **M (SD) or %** | ***p* value** |
| **Neurodevelopment (Battelle scores)** |  |  |  |  |  |  |  |
| Social | 3,827 | 4 – 28 | 25.41 (2.16) | 24.78 (2.51) | 23.73 (3.56) | 23.89 (3.13) | **0.008** |
| Adaptative | 3,821 | 3 – 28 | 26.39 (1.81) | 26.27 (1.64) | 25.47 (3.25) | 26.12 (1.78) | **0.008** |
| Motor | 3,750 | 10 – 28 | 23.01 (2.30) | 22.98 (2.28) | 22.30 (2.54) | 22.49 (2.45) | **0.008** |
| Communication | 3,776 | 1 – 24 | 19.16 (2.53) | 18.34 (2.35) | 17.52 (3.53) | 18.11 (2.89) | **0.008** |
| Cognitive | 3,783 | 0 – 24 | 20.04 (2.57) | 19.28 (2.93) | 18.60 (3.35) | 18.99 (3.07) | **0.008** |
| **Language** |  |  |  |  |  |  |  |
| Expressive | 3,768 | 0 – 93 | 54.28 (19.56) | 46.73 (19.58) | 43.82 (21.99) | 42.01 (20.27) | **0.008** |
| Receptive | 3,835 | 0 – 33 | 25.27 (5.09) | 23.04 (5.77) | 22.28 (5.77) | 21.49 (6.56) | **0.008** |
| **Executive functions** |  |  |  |  |  |  |  |
| Self-control (interviewer rating) | 3,893 | 4 – 12 | 10.01 (2.45) | 9.19 (2.89) | 9.01 (2.88) | 8.49 (2.97) | **0.008** |
| Gratification delay (Marshmallow task) | 3,433 | 0 – 210 | 179.47 (63.91) | 162.80 (75.10) | 158.01 (77.52) | 162.37 (74.66) | **0.008** |
| Impulse control (EYT task) | 2,854 | 0 – 1 | 0.36 (0.18) | 0.32 (0.17) | 0.32 (0.18) | 0.30 (0.17) | **0.008** |
| Attention Shifting (EYT task) | 3,806 | 0 / 1 | 14.2% | 11.1% | 9.2% | 9.1% | 0.164 |
| **Emotion recognition/Theory of mind** |  |  |  |  |  |  |  |
| Emotion recognition | 3,556 | 0 – 5 | 2.28 (1.06) | 1.97 (1.12) | 1.86 (1.07) | 1.55 (0.99) | **0.008** |
| Theory of Mind | 3,778 | 0 – 3 | 1.72 (0.84) | 1.67 (0.86) | 1.60 (0.84) | 1.53 (0.85) | 0.156 |
| **Social information processing** |  |  |  |  |  |  |  |
| Hostile attribution bias | 3,229 | 0 / 1 | 70.4% | 68.5% | 70.0% | 70.0% | 0.961 |
| Aggressive response generation | 2,969 | 0 / 1 | 12.5% | 13.4% | 16.0% | 11.4% | 0.961 |
| Competent assessment score | 3,060 | 0 - 9 | 5.92 (1.93) | 6.03 (1.92) | 5.79 (1.83) | 6.00 (1.79) | 0.961 |
| **Prosocial behaviour** |  |  |  |  |  |  |  |
| Empathy-altruism (Help Task) | 3,706 | 0 – 3 | 1.26 (1.25) | 1.19 (1.26) | 1.04 (1.22) | 1.15 (1.24) | 0.156 |
| Altruism (Dictator game) | 3,827 | 0 – 10 | 3.49 (2.67) | 3.06 (2.51) | 3.42 (2.90) | 3.01 (2.37) | 0.164 |
| Prosocial behaviour | 3,997 | 0 – 10 | 8.99 (1.24) | 8.20 (1.61) | 7.25 (1.93) | 6.62 (2.42) | **0.008** |

***Note.*** *Range of observed values in the current sample. ANOVA for count and continuous variables. Chi-square test for dichotomous variables. *p* values adjusted for multiple comparisons by Benjamini-Hochberg procedure.

Table S6. Associations between groups of higher/lower callous unemotional (CU) traits (cut-off ≥14) and conduct problems (CP) (cut-off ≥6) and children's psychosocial characteristics, adjusting for covariates.

|  | **Higher**  **CP-only** | **Higher**  **CU-only** | **Higher**  **CP+CU** |  |
| --- | --- | --- | --- | --- |
|  | **Adjusted IRR or β* (95% CI)^#^**  (Reference category: Typically developing) | | | ***p* value** |
| **Neurodevelopment (Battelle scores)** |  |  |  |  |
| Social | **0.99 (0.97, 1.00)** | **0.94 (0.93, 0.96)** | **0.95 (0.93, 0.98)** | **0.010** |
| Adaptative | 1.00 (0.99, 1.00) | **0.97 (0.95, 0.98)** | 0.99 (0.98, 1.00) | **0.010** |
| Motor | 1.01 (0.99, 1.02) | **0.98 (0.96, 0.99)** | 0.99 (0.97, 1.01) | **0.045** |
| Communication | **0.97 (0.96, 0.99)** | **0.93 (0.91, 0.95)** | **0.97 (0.94, 1.00)** | **0.010** |
| Cognitive | **0.98 (0.96, 1.00)** | **0.94 (0.92, 0.96)** | **0.97 (0.94, 1.00)** | **0.010** |
| **Language** |  |  |  |  |
| Expressive | **0.94 (0.89, 0.99)** | **0.88 (0.83, 0.93)** | **0.87 (0.80, 0.95)** | **0.010** |
| Receptive | **0.95 (0.92, 0.98)** | **0.92 (0.89, 0.95)** | **0.90 (0.85, 0.95)** | **0.010** |
| **Executive Functions** |  |  |  |  |
| Self-control (interviewer rating) | **0.94 (0.90, 0.98)** | **0.92 (0.88, 0.95)** | **0.87 (0.82, 0.93)** | **0.010** |
| Gratification delay (Marshmallow task) | **0.92 (0.86, 0.98)** | **0.89 (0.84, 0.95)** | 0.92 (0.84, 1.01) | **0.010** |
| Impulse control (EYT task)* | -0.02 (-0.05, 0.01) | -0.02 (-0.04, 0.01) | -0.04 (-0.07, 0.00) | 0.936 |
| Attention Shifting (EYT task)† | 1.02 (0.69, 1.52) | 0.84 (0.57, 1.23) | 0.90 (0.50, 1.63) | 0.937 |
| **Emotion recognition/Theory of mind** |  |  |  |  |
| Emotion Recognition | **0.95 (0.88, 1.03)** | **0.89 (0.83, 0.96)** | **0.77 (0.68, 0.87)** | **0.010** |
| Theory of Mind | 1.00 (0.93, 1.08) | 0.95 (0.89, 1.02) | 0.92 (0.83, 1.03) | 0.937 |
| **Social Information Processing** |  |  |  |  |
| Hostile attribution bias† | 0.97 (0.87, 1.07) | 0.99 (0.90, 1.08) | 0.99 (0.86, 1.14) | 0.937 |
| Aggressive response generation† | 1.04 (0.70, 1.56) | 1.26 (0.90, 1.77) | 0.86 (0.45, 1.64) | 0.937 |
| Competent assessment score | 1.02 (0.97, 1.07) | 0.98 (0.94, 1.03) | 1.01 (0.95, 1.08) | 0.937 |
| **Prosocial behaviour** |  |  |  |  |
| Empathy-altruism (Help Task) | 0.97 (0.83, 1.13) | **0.85 (0.73, 0.98)** | 0.95 (0.77, 1.17) | 0.936 |
| Altruism (Dictator game) | 0.90 (0.80, 1.01) | 1.00 (0.90, 1.12) | 0.89 (0.76, 1.03) | 0.936 |
| Prosocial behaviour | **0.92 (0.89, 0.94)** | **0.81 (0.78, 0.83)** | **0.74 (0.69, 0.79)** | **0.010** |
| ***Note.*** IRR: Incidence Rate Ratio from Poisson Regression (representing Prevalence Ratios for dichotomous variables†, based on robust error variance). *β from Linear Regression. Results shown in bold p<0.05 or 95% CI does not include the null value. *p* values adjusted for multiple comparisons by Benjamini-Hochberg procedure. Adjusted for sex, maternal age, maternal schooling and family income. | | | | |

Table S7. Psychosocial characteristics of children with higher/lower callous unemotional (CU) traits and conduct problems (CP), stratified by sex.

|  | **BOYS** | | | | | | **GIRLS** | | | | |  |
| --- | --- | --- | --- | --- | --- | --- | --- | --- | --- | --- | --- | --- |
|  | **Typically developing** | **Higher**  **CP-only** | **Higher**  **CU-only** | **Higher**  **CP+CU** |  |  | **Typically developing** | **Higher**  **CP-only** | **Higher**  **CU-only** | **Higher**  **CP+CU** |  | |
|  | **M (SD) OR %** | **M (SD) OR %** | **M (SD) OR %** | **M (SD) OR %** | ***p* value** |  | **M (SD) OR %** | **M (SD) OR %** | **M (SD) OR %** | **M (SD) OR %** | ***p* value** | |
| **Neurodevelopment  (Battelle scores)** |  |  |  |  |  |  |  |  |  |  |  | |
| Social | 25.32 (2.21) | 24.87 (2.57) | 24.15 (3.26) | 24.28 (3.20) | **0.010** |  | 25.90 (1.66) | 25.49 (1.86) | 24.89 (2.34) | 24.83 (2.46) | **0.010** | |
| Adaptative | 26.24 (1.84) | 26.04 (2.26) | 25.66 (2.76) | 25.91 (2.05) | **0.010** |  | 26.67 (1.56) | 26.56 (1.55) | 26.36 (2.27) | 26.37 (1.97) | 0.110 | |
| Motor | 22.59 (2.34) | 22.57 (2.23) | 22.11 (2.41) | 22.54 (2.41) | 0.113 |  | 23.55 (2.15) | 23.32 (2.21) | 22.98 (2.42) | 23.16 (2.42) | **0.010** | |
| Communication | 19.11 (2.68) | 18.41 (2.86) | 18.02 (3.39) | 18.16 (3.06) | **0.010** |  | 19.58 (2.23) | 19.00 (1.95) | 18.91 (2.64) | 18.68 (2.42) | **0.010** | |
| Cognitive | 19.95 (2.52) | 19.39 (3.09) | 18.91 (3.12) | 18.97 (3.08) | **0.010** |  | 20.57 (2.38) | 19.87 (2.35) | 19.60 (2.85) | 19.63 (2.53) | **0.010** | |
| **Language** |  |  |  |  |  |  |  |  |  |  |  | |
| Expressive | 54.66 (20.71) | 48.14 (21.25) | 47.92 (20.17) | 46.43 (21.57) | **0.010** |  | 57.48 (17.76) | 53.81 (17.60) | 48.43 (20.10) | 47.72 (19.65) | **0.010** | |
| Receptive | 24.79 (5.08) | 22.93 (5.85) | 22.73 (5.60) | 22.63 (6.05) | **0.010** |  | 26.69 (4.34) | 25.87 (4.85) | 24.55 (5.45) | 23.71 (5.52) | **0.010** | |
| **Executive Functions** |  |  |  |  |  |  |  |  |  |  |  | |
| Self-control | 9.89 (2.43) | 9.09 (2.86) | 9.33 (2.77) | 8.79 (2.96) | **0.010** |  | 10.51 (2.13) | 9.83 (2.68) | 9.97 (2.44) | 9.65 (2.62) | **0.010** | |
| Gratification delay | 178.90 (64.77) | 164.02 (75.62) | 160.92 (77.80) | 159.98 (75.35) | **0.010** |  | 188.28 (53.41) | 178.51 (66.23) | 173.03 (71.05) | 171.43 (69.61) | **0.010** | |
| Impulse control | 0.35 (0.17) | 0.32 (0.16) | 0.32 (0.17) | 0.32 (0.18) | 0.318 |  | 0.38 (0.19) | 0.35 (0.17) | 0.34 (0.20) | 0.35 (0.18) | 0.110 | |
| Attention Shifting | 12.8% | 6.6% | 7.5% | 10.6% | **0.054** |  | 18.2% | 15.6% | 15.8% | 10.4% | 0.110 | |
| **Emotion recognition/ Theory of mind** |  |  |  |  |  |  |  |  |  |  |  | |
| Emotion recognition | 2.29 (1.07) | 2.05 (1.12) | 1.95 (1.10) | 1.90 (1.02) | **0.010** |  | 2.48.(1.02) | 2.18 (1.01) | 1.89 (1.03) | 1.97 (1.12) | **0.010** | |
| Theory of mind | 1.70 (0.82) | 1.56 (0.86) | 1.55 (0.84) | 1.62 (0.81) | 0.072 |  | 1.78 (0.85) | 1.73 (0.85) | 1.66 (0.89) | 1.77 (0.86) | 0.890 | |
| **Social Information Processing** |  |  |  |  |  |  |  |  |  |  |  | |
| Hostile attribution bias | 73.1% | 69.0% | 66.1% | 67.2% | 0.345 |  | 70.0% | 73.0% | 66.8% | 71.4% | 0.890 | |
| Aggressive response generation | 11.2% | 17.6% | 12.9% | 12.8% | 0.345 |  | 12.5% | 14.6% | 12.9% | 13.1% | 0.890 | |
| Competent assessment score | 5.94 (1.91) | 5.85 (1.92) | 5.76 (2.03) | 5.85 (1.89) | 0.639 |  | 6.01 (1.94) | 5.72 (1.90) | 5.76 (1.80) | 6.21 (1.83) | 0.110 | |
| **Prosocial behaviour** |  |  |  |  |  |  |  |  |  |  |  | |
| Empathy-altruism (Help Task) | 1.23 (1.25) | 1.19 (1.20) | 1.07 (1.21) | 1.12 (1.21) | 0.400 |  | 1.38 (1.26) | 1.35 (1.27) | 1.11 (1.24) | 1.15 (1.25) | **0.036** | |
| Altruism (Dictator game) | 3.48 (2.81) | 3.06 (2.88) | 3.28 (2.94) | 3.21 (2.73) | 0.345 |  | 3.61 (2.52) | 3.55 (2.44) | 3.57 (2.59) | 3.37 (2.48) | 0.890 | |
| Prosocial behaviour | 9.18 (1.12) | 8.77 (1.26) | 8.11 (1.69) | 7.46 (1.98) | **0.010** |  | 9.26 (1.02) | 8.98 (1.15) | 8.22 (1.68) | 7.79 (1.88) | **0.010** | |

***Note.*** ANOVA for count and continuous variables. Chi-square test for dichotomous variables. *p* values adjusted for multiple comparisons by Benjamini-Hochberg procedure.

Table S8. Associations between groups of higher/lower callous unemotional (CU) traits and conduct problems (CP) and children’s psychosocial characteristics stratifying by sex and adjusting for covariates, and *p* value of interaction term between group and sex.

|  | **BOYS** | | |  |  | **GIRLS** | | |  | **Interaction term** |
| --- | --- | --- | --- | --- | --- | --- | --- | --- | --- | --- |
|  | **Higher**  **CP-only** | **Higher**  **CU-only** | **Higher**  **CP+CU** | ***p* value** |  | **Higher**  **CP-only** | **Higher**  **CU-only** | **Higher**  **CP+CU** | ***p* value** |  |
|  | **Adjusted IRR or β* (95% CI)**  Reference category: Typically developing | | | |  | **Adjusted IRR or β* (95% CI)**  Reference category: Typically developing | | | | ***p* value** |
| **Neurodevelopment  (Battelle scores)** |  |  |  |  |  |  |  |  |  |  |
| Social | 0.99 (0.98, 1.00) | **0.96 (0.94, 0.98)** | **0.97 (0.95, 0.98)** | **0.011** |  | 0.99 (0.98, 1.00) | **0.97 (0.96, 0.98)** | **0.97 (0.96, 0.98)** | **0.013** | **0.010** |
| Adaptative | 0.99 (0.98, 1.00) | **0.98 (0.96, 0.99)** | **0.98 (0.98, 0.99)** | **0.011** |  | 1.00 (0.99, 1.00) | 0.99 (0.98, 1.00) | 0.99 (0.98, 1.00) | 0.600 | **0.010** |
| Motor | 1.00 (0.99, 1.02) | **0.98 (0.97, 0.99)** | 1.00 (0.98, 1.01) | 0.272 |  | 1.00 (0.98, 1.01) | 0.98 (0.97, 1.00) | 0.99 (0.98, 1.01) | 0.784 | 0.117 |
| Communication | 0.98 (0.96, 1.00) | **0.95 (0.93, 0.98)** | **0.96 (0.94, 0.98)** | **0.011** |  | 0.98 (0.97, 1.00) | 0.98 (0.96, 1.00) | **0.98 (0.96, 0.99)** | 0.077 | **0.010** |
| Cognitive | 0.98 (0.96, 1.00) | **0.96 (0.94, 0.98)** | **0.96 (0.94, 0.98)** | **0.011** |  | **0.98 (0.96, 0.99)** | **0.97 (0.95, 0.99)** | **0.97 (0.96, 0.99)** | **0.013** | **0.010** |
| **Language** |  |  |  |  |  |  |  |  |  |  |
| Expressive | **0.93 (0.88, 0.98)** | **0.94 (0.89, 0.99)** | **0.92 (0.87, 0.97)** | **0.011** |  | 1.00 (0.96, 1.04) | **0.91 (0.86, 0.96)** | **0.92 (0.87, 0.96)** | **0.013** | **0.010** |
| Receptive | **0.95 (0.92, 0.98)** | **0.95 (0.92, 0.97)** | **0.95 (0.92, 0.98)** | **0.011** |  | 1.00 (0.98, 1.02) | **0.95 (0.93, 0.98)** | **0.93 (0.91, 0.96)** | **0.013** | **0.010** |
| **Executive Functions** |  |  |  |  |  |  |  |  |  |  |
| Self-control | **0.92 (0.89, 0.96)** | **0.95 (0.91, 0.98)** | **0.90 (0.86, 0.93)** | **0.011** |  | **0.95 (0.92, 0.98)** | 0.96 (0.93, 1.00) | **0.94 (0.91, 0.97)** | **0.013** | **0.010** |
| Gratification delay | **0.92 (0.87, 0.98)** | **0.90 (0.85, 0.96)** | **0.90 (0.85, 0.95)** | **0.011** |  | 0.96 (0.91, 1.00) | **0.93 (0.87, 0.98)** | **0.92 (0.87, 0.98)** | **0.036** | **0.010** |
| Impulse control* | -0.01 (-0.04, 0.01) | -0.02 (-0.04, 0.01) | -0.01 (-0.04, 0.01) | 0.646 |  | -0.01 (-0.04, 0.01) | -0.02 (-0.05, 0.01) | -0.01 (-0.04, 0.02) | 0.867 | 0.595 |
| Attention Shifting† | **0.60 (0.37, 0.96)** | 0.69 (0.45, 1.06) | 1.00 (0.70, 1.41) | 0.438 |  | 1.03 (0.76, 1.39) | 1.07 (0.78, 1.47) | 0.76 (0.52, 1.11) | 0.867 | 0.588 |
| **Emotion recognition/ Theory of mind** |  |  |  |  |  |  |  |  |  |  |
| Emotion recognition | 0.95 (0.88, 1.02) | **0.91 (0.85, 0.98)** | **0.90 (0.84, 0.96)** | **0.030** |  | 0.94 (0.89, 1.00) | **0.83 (0.77, 0.89)** | **0.88 (0.82, 0.95)** | **0.013** | **0.010** |
| Theory of mind | 0.92 (0.86, 1.00) | **0.92 (0.85, 0.98)** | 0.96 (0.90, 1.02) | 0.270 |  | 0.99 (0.93, 1.06) | 0.96 (0.89, 1.03) | 1.02 (0.96, 1.09) | 0.867 | 0.588 |
| **Social Information Processing** |  |  |  |  |  |  |  |  |  |  |
| Hostile attribution bias† | 0.94 (0.85, 1.03) | **0.89 (0.81, 0.99)** | **0.91 (0.83, 0.99)** | 0.336 |  | 1.04 (0.95, 1.14) | 0.95 (0.86, 1.06) | 1.02 (0.92, 1.12) | 0.867 | 0.588 |
| Aggressive response generation† | 1.52 (1.07, 2.18) | 1.10 (0.75, 1.62) | 1.10 (0.74, 1.61) | 0.646 |  | 1.17 (0.81, 1.70) | 1.04 (0.70, 1.56) | 1.05 (0.70, 1.59) | 0.867 | 0.595 |
| Competent assessment score | 0.98 (0.94, 1.03) | 0.97 (0.92, 1.02) | 0.98 (0.94, 1.03) | 0.646 |  | 0.95 (0.91, 1.00) | 0.96 (0.91, 1.00) | 1.03 (0.99, 1.08) | 0.170 | 0.448 |
| **Prosocial behaviour** |  |  |  |  |  |  |  |  |  |  |
| Empathy-altruism (Help Task) | 0.98 (0.86, 1.13) | 0.89 (0.77, 1.03) | 0.93 (0.82, 1.07) | 0.646 |  | 1.00 (0.88, 1.14) | **0.82 (0.70, 0.96)** | **0.86 (0.74, 0.99)** | 0.180 | 0.368 |
| Altruism (Dictator game) | 0.90 (0.79, 1.01) | 0.96 (0.89, 1.08) | 0.94 (0.85, 1.04) | 0.646 |  | 1.00 (0.91, 1.09) | 1.01 (0.91, 1.11) | 0.96 (0.86, 1.06) | 0.867 | 0.595 |
| Prosocial behaviour | **0.96 (0.94, 0.97)** | **0.88 (0.86, 0.90)** | **0.81 (0.79, 0.84)** | **0.011** |  | **0.97 (0.95, 0.99)** | **0.89 (0.87, 0.91)** | **0.84 (0.82, 0.87)** | **0.013** | **0.010** |

***Note.*** IRR: Incidence Rate Ratio from Poisson Regression (representing Prevalence Ratios for dichotomous variables†, based on robust error variance). *β from Linear Regression. Results shown in bold p<0.05 or 95% CI does not include the null value. *p* values adjusted for multiple comparisons by Benjamini-Hochberg procedure. Adjusted for maternal age, maternal schooling and family income.

REFERENCES

Barros, A. J. D., Matijasevich, A., Santos, I. S., & Halpern, R. (2010). Child development in a birth cohort: Effect of child stimulation is stronger in less educated mothers. *International Journal of Epidemiology*, *39*(1), 285–294. https://doi.org/10.1093/ije/dyp272

Benenson, J. F., Pascoe, J., & Radmore, N. (2007). Children’s altruistic behavior in the dictator game. *Evolution and Human Behavior*, *28*(3), 168–175. https://doi.org/10.1016/j.evolhumbehav.2006.10.003

Buttelmann, D., Carpenter, M., & Tomasello, M. (2009). Eighteen-month-old infants show false belief understanding in an active helping paradigm. *Cognition*, *112*(2), 337–342. https://doi.org/10.1016/j.cognition.2009.05.006

Capovilla, F., Negrão, V., & Damázio, M. (2011). *Teste de Vocabulário Auditivo e Teste de Vocabulário Expressivo: validados e normatizados para o desenvolvimento da compreensão da fala dos 18 meses aos 6 anos de idade.* (First Edit). Memnon Edições Científicas LTDA.

Caspi, A., Henry, B., McGee, R. O., Moffitt, T. E., & Silva, P. A. (1995). Temperamental origins of child and adolescent behavior problems: from age three to age fifteen. *Child Development*, *66*(1), 55–68. https://doi.org/10.1111/j.1467-8624.1995.tb00855.x

Fujino, H., Fukushima, K., & Fujiyoshi, A. (2017). Theory of mind and language development in Japanese children with hearing loss. *International Journal of Pediatric Otorhinolaryngology*, *96*, 77–83. https://doi.org/10.1016/j.ijporl.2017.03.005

Goodman, R. (1999). The extended version of the Strengths and Difficulties Questionnaire as a guide to child psychiatric caseness and consequent burden. *Journal of Child Psychology and Psychiatry and Allied Disciplines*, *40*(5), 791–799. https://doi.org/10.1017/S0021963099004096

Howard, S. J., & Melhuish, E. (2017). An Early Years Toolbox for Assessing Early Executive Function, Language, Self-Regulation, and Social Development: Validity, Reliability, and Preliminary Norms. *Journal of Psychoeducational Assessment*, *35*(3), 255–275. https://doi.org/10.1177/0734282916633009

Viding, E., Blair, R. J. R., Moffitt, T. E., & Plomin, R. (2005). Evidence for substantial genetic risk for psychopathy in 7-years-olds. *Journal of Child Psychology and Psychiatry and Allied Disciplines*, *46*(6), 592–597. https://doi.org/10.1111/j.1469-7610.2004.00393.x

Zelazo, P. D. (2006). The Dimensional Change Card Sort (DCCS): A method of assessing executive function in children. *Nature Protocols*, *1*(1), 297–301. https://doi.org/10.1038/nprot.2006.46

Ziv, Y., & Sorongon, A. (2011). Social information processing in preschool children: Relations to sociodemographic risk and problem behavior. *Journal of Experimental Child Psychology*, *109*(4), 412–429. https://doi.org/10.1016/J.JECP.2011.02.009
